# Analysis of trends, recurrences, severity and frequency of droughts using standardised precipitation index: Case of OR Tambo District Municipality, Eastern Cape, South Africa

**DOI:** 10.4102/jamba.v14i1.1147

**Published:** 2022-02-25

**Authors:** Melezwa Nkamisa, Simbarashe Ndhleve, Motebang D.V. Nakin, Asabonga Mngeni, Hlekani M. Kabiti

**Affiliations:** 1 Department of Biological and Environmental Science, Faculty of Natural Sciences, Walter Sisulu University, Mthatha, South Africa; 2 Risk and Vulnerability Science Centre, Walter Sisulu University, Mthatha, South Africa

**Keywords:** agricultural drought, climate change, hydrological drought, meteorological drought monitor, standardised precipitation index

## Abstract

South Africa is susceptible to droughts. However, little documentation exists on drought occurrence in South Africa at national, provincial and municipal administrative boundaries. This study profiles hydrological drought in OR Tambo District Municipality from 1998 to 2018, computing frequency, severity and intensity in order to show areas of high vulnerability. Data used were obtained from South African Weather Services. Standardised precipitation index (SPI) was calculated using the Meteorological Drought Monitor (MDM) software. Results showed a wide variation in monthly precipitation throughout the year. Coastal areas receive higher rainfall than inland municipalities. The study revealed that Nyandeni experienced the highest drought frequency of 62%, Mhlontlo (58%), King Sabatha Dalindyebo Municipality (57%), Ngquza Hill (55%) and Port St Johns Municipality showing the least at 52%. Hydrological drought severity frequency and duration varied between seven days and nine weeks. Drought intensity class exposed the annual average intensity for the five local municipalities represented as follows: KSDM (–0.71), PSJM (–0.99), Ngquza Hill (–0.81), Nyandeni (–0.71) and Mhlontlo (–0.62). The longest drought duration across OR Tambo was experienced in 2014 with durations varying from 3 to 11 weeks across the municipalities. OR Tambo District Municipality is susceptible to hydrological droughts and the extent varies across local municipalities. Results could be used for both adaptation planning and mitigating the impacts of future droughts. In addition, they could assist in guiding allocation of drought relief resources in ways that prioritise drought prone and vulnerable municipality.

## Introduction

Agriculture is an important sector in South Africa. It has remained a significant provider of employment in the rural areas, and a major earner of foreign exchange (Bates 2014). In South Africa, economic growth in rural areas, where more than 70% of the population is regarded as poor but has access to abundant land, is dependent on agricultural production (Mabogunje 2015). About 70% of agricultural output is used as intermediate products in other sectors and this makes it a crucial sector with several multiplier effects on the rest of the economy (McCombie & Thirlwall 2016). The sector’s interconnectedness with the larger economy cannot be overemphasised. Agriculture is susceptible to droughts and these droughts have multiple socio-economic effects (Gilmore 2017). Droughts are considered major natural hazards causing destructive impact on livelihoods, the environment as well as the economies (Alexander 2017). They have both direct and indirect socio-economic impacts and their effects are more damaging for economies driven primarily by agriculture. The nexus amongst droughts and climate change, agriculture, food security and poverty reduction stands out prominently in the current theoretical and empirical debates on economic development (Gilmore 2017). Droughts negatively impact agricultural production, thereby affecting the four dimensions of food security, that is, availability, stability, access and utilisation (Cheeseman 2016). Studies from many developing countries strongly concur that rural economic growth and wide-spread poverty reduction require increased production in agriculture (McGlade et al. 2019). Many studies concur that droughts negatively impact agricultural production and efforts to reduce poverty (Udmale 2014).

South Africa is a naturally dry country that is highly vulnerable to droughts and also a major producer of agricultural goods in Southern Africa (Adams 2014). It is self-sufficient in a range of food commodities and usually produces exportable surpluses (Gilmore 2017). Southern African countries, such as Namibia, Botswana, Zimbabwe, Lesotho, Zambia and Mozambique, significantly rely on agricultural imports from South Africa. Droughts have multiple effects on agriculture ranging from crop losses, lower yields in crop and livestock production, increased livestock deaths, increases in insect infestation and plant and animal diseases, damage to fish habitat, forest and range fires, land degradation and soil erosion (Udmale 2014). Furthermore, there is a compelling body of knowledge that links droughts to other epidemics like famine, diseases and land degradation globally (Adams 2014). Adams (2014) further stated that droughts impact on human health through increased risk of food and water shortages, increased risk of malnutrition and higher risk of water and food borne diseases. Thus, drought represents a constant threat to health, food security and livelihoods (Davies 2016). Despite being a major regional player in agriculture, droughts are regular and recurrent in South Africa (Davis & Vincent 2017). Droughts have a recurrent characteristic feature; and this is especially the case for South Africa because of its highly variable climate (Tauma et al. 2015).

South Africa’s annual average rainfall is approximately 450 mm and that makes the country prone to recurrent droughts (Hirooka et al. 2019). Drought periods can be characterised from a few hours (short-term) to millennia (long-term) and there are four categories, namely, meteorological drought, hydrological drought, agricultural drought and socio-economic drought (Botai et al. 2016).

Hydrological drought is associated with the deficiency of water on the surface or subsurface because of shortfall in precipitation and is characterised by persistent reduction in runoff (Gu et al. 2020). Meteorological drought implies rainfall deficiency where the precipitation is reduced by 25% from normal in any given area. It is characterised by lack of precipitation over an extended period and is mainly determined by climatic conditions and atmospheric circulations (Alvala et al. 2017). It is defined usually on the basis of the degree of dryness in comparison to some ‘normal’ or average amount and the duration of the dry period and its definitions are considered as region specific (Miralles et al. 2019). Agricultural drought links various meteorological or hydrological characteristics to agricultural impacts. It focuses on precipitation shortages, and differences amongst actual potential evapotranspiration, soil, soil water deficit and reduced ground water and reservoir levels help to learn more on agriculture (Bae et al. 2019; Miralles et al. 2019). Agricultural drought is characterised by reduction of soil moisture following reduction of precipitation, runoff and soil moisture. This is usually associated with the shortage of water supply for population, livestock, industry, ecology, environment may become much more serious. Socio-economic drought is associated with the demand and supply aspect of economic goods together with the elements of meteorological, hydrological and agricultural drought. Primarily, socio-economic drought is influenced by both natural and anthropogenic systems and is affected mostly by human activities (Pedro-Monzonis et al. 2015). Socio-economic drought occurs when there is reduction of precipitation, runoff and soil moisture, and there is shortage of water supply for population, livestock, industry, ecology and environment. Socio-economic definitions of drought associate the supply and demand of some economic good with elements of meteorological, hydrological and agricultural drought (Alvala et al. 2017).

The effects of drought are not uniform with regard to time and place as their nature is indicated by precipitation, temperature, stream flow, groundwater and reservoir levels, soil moisture and snowpack (Botai et al. 2016). In addition, droughts are triggered by different factors and so are their frequency and intensity. One school of thought argued that some of the problems caused by drought are difficult to avoid whilst some are avoidable through proper planning and effective drought responses (Staupe-Delgado & Kruke 2017). Thus, the need for comprehensive information aided by a comprehensive research that seeks to provide baseline information on drought cannot be overemphasised (Mwangi 2016). There is very little documentation on the incidences of drought in South Africa at national level and at its various administrative boundaries that can aid proper planning. South Africa is likely to experience more frequent and severe droughts in future (Hirooka et al. 2019). It is highly probable that increased climate volatility will result in increased frequency and intensity of droughts. However, to date, very little research has been performed to profile droughts in South Africa. Profiling droughts has multiple benefits including identifying the most vulnerable areas for the purpose of improving monitoring, planning, raising awareness and interventions. This could also help in the delineation of major areas facing drought risk for effective management plans formulation by government authorities. Therefore, this study aims to establish baseline information on the frequency and intensity of droughts in South Africa. The general intention of this study is to comprehensively profile all the droughts that occurred in OR Tambo District Municipality (ORTDM) during the period between 1998 and 2018. The study specifically focuses on computing the frequency, severity and intensity of the droughts in South Africa’s poorest province, Eastern Cape. This information could be used in the development of a comprehensive and flexible drought management strategy to effectively reduce the impact of future droughts.

## Materials and methods

### Study area

**FIGURE 1:**
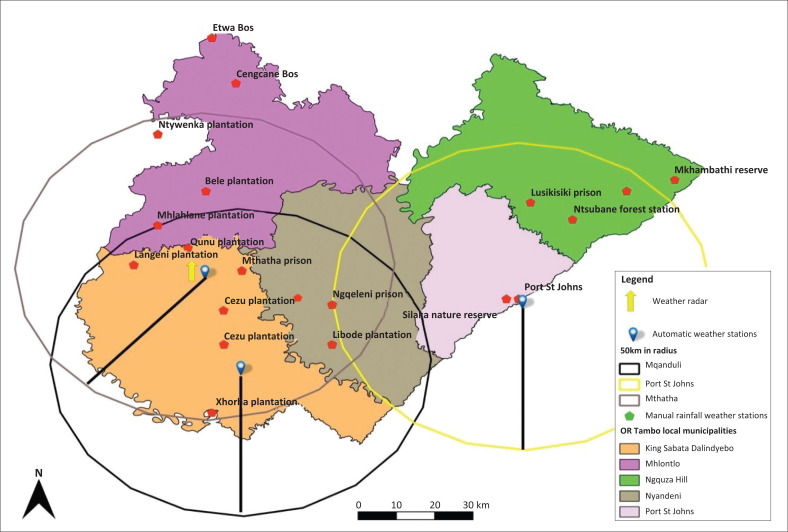
Distribution of weather stations in OR Tambo District Municipality.

The ORTDM occupies the eastern coastal portion of the Eastern Cape province, South Africa. The district lies along the coastline of the Indian Ocean stretching for up to 160 km (Morgan 2017). The district extends over a geographical area of 15946.84 km² and incorporates five local municipalities, referred to by Figure 1 (Morgan 2017). ORTDM lies between the coordinates of 32°46’31”S and 21°23’29”E (Mlanjeni 2014). OR Tambo is classified as a Category C2 municipality, indicating a largely rural character and low urbanisation rate. In addition to agriculture, the other economic sectors are community services, trade, finance, transport, manufacturing and construction (Null 2018). Its suitable terrain and many river valleys provide irrigable land, abundant water resources, large tracts of grazing land, suitable pasture species for stock grazing, and a large number of stock owned by rural communities (Davies 2014). The district has the richest natural resources and the most fertile soils and favourable climatic conditions. Agricultural practices are intense although climate dependent (Thomas 2014). These have diverse vegetation types ranging from grasslands, thicket, forests and bushveld (Munn 2018). Mlanjeni (2014) notes that drought incidents negatively impact agricultural production of the OR Tambo district and contribute to food insecurity. The OR Tambo district receives plenty of rainfall and increased humidity during summer. Winters are colder especially in inland areas. The district’s climatic conditions are moderate to high rainfall areas, mainly along its sub-tropical coast and in pockets of mountainous areas (Narloch & Bangalore 2018). The climatic conditions of OR Tambo district have warm, temperate, predominantly frost-free conditions. The OR Tambo district enjoys a high level of annual sunshine, and in summer, temperatures range from 16 ºC to 28 ºC whilst winter temperatures range from 7 ºC to 20 ºC. Winter months fall between April and August whilst summer temperatures are usually highest between November and April (Slattery 1998). The people in the OR Tambo district enjoy four seasons of the year namely, summer, winter, spring and autumn and they are characterised by different weather conditions ranging from hot, to cool, mild, windy and cold conditions.

### Data sources

Data used on this study were obtained at South African Weather Services (SAWS) in Pretoria. The study employed daily, monthly and annual precipitation data gathered from the SAWS for the period 1998 to 2018 and recorded the data using both automatic and manual weather stations located across ORTDM. There are a number of weather stations in the ORTDM. Before 1979, the municipality had 17 operating weather stations to assist in weather recording and forecasting. Advances in technology led to the addition of three automatic weather stations in ORTDM. The automatic weather stations are located in Port St Johns and King Sabatha Dalindyebo municipalities, see Figure 1. According to Mlanjeni (2014), manual weather stations are not advanced because they had a majority of disadvantages such as missing data and lacked coherence. Advances in technology and the introduction of automatic weather stations became an alternative, which is a more reliable solution. Table 1 shows all the weather stations in ORTDM, their spatial locations and type categorised into automatic and manual weather stations. Figure 1 shows the map for ORTDM, the local municipalities and the weather stations, both automatic and manual weather stations.

**TABLE 1:** Rainfall weather stations in OR Tambo District Municipality with Global Positioning System (GPS) co-ordinates.

Weather station	Latitude	Longitude	Type of weather station
Mthatha prison	31˚34′59.46″S	28˚26′24.52″E	Automatic
Baziya plantation	33˚34′08.98″S	28˚26′00.61″E	Manual
Qunu plantation	31˚46′49.54″S	28˚37′24.81″E	Manual
Langeni plantation	31˚29′7.61″S	28˚28′51.95″E	Manual
Libode plantation	31˚32′4.15″S	29˚1′10.33″E	Manual
Cezu plantation	31˚46′31.80″S	28˚43′44.86″E	Manual
Ngqeleni prison	31˚40′12.00″S	29˚1′40.80″E	Manual
Silaka nature reserve	31˚39′9.50″S	29˚39′9.50″E	Manual
Port St Johns municipality	31˚38′23.27″S	29˚32′33.57″E	Automatic
Mhlahlane plantation	31˚25′15.73″S	28˚32′38.53″E	Manual
Ntywenka plantation	31˚9′49.92″S	28˚32′30.72″E	Manual
Bele plantation	31˚19′36.91″S	28˚40′12.28″E	Manual
Lusikisiki prison	31˚21′58.00″S	29˚34′21.28″E	Manual
Xhorha plantation	31˚58′15.52″S	28˚41′23.19″E	Automatic
Ntsubane forest station	31˚24′24.72″S	29˚41′48.59″E	Manual
Mkhambathi Reserve	31˚17′42.40″S	29˚58′47.57″E	Manual
Etwa Bos	30˚17′43.31″E	29˚50′17.25″E	Manual
Cengcane Bos	31˚00′37.75″S	28˚45′41.57″E	Manual

*Source*: Google Earth.

### Data analysis

Rainfall data were used to compute average annual precipitation for the period 1998 to 2018, determining periods and areas with below normal and above normal average precipitation. Standardised precipitation index (SPI) was used to compute drought frequency, severity and intensity to expose the high drought risk areas. Various indices have been developed to assess the onset, severity, frequency, intensity and end of droughts (Mahlalela, Blamey & Reason 2018). The selection and application of these methods are based on the anticipated objectives, nature of the indicator, local conditions, data availability and data validity (Gerwin et al. 2018; Gqwede 2018; Maza et al. 2019). This study employed the SPI because of its popularity and ability to synthesise long-term data records of precipitation. This study’s rainfall data spanned over 20 years. A number of studies employed the SPI and commended the index (Gqwede 2018; Sprecher 2017). In addition to being widely recommended, this study’s choice of SPI was also influenced by the nature of the available data.

In order for the results to be precise, understandable and presentable, the Meteorological Drought Monitor (MDM) software programme was used to compute the SPI values for the moving average at 3 months, 6 months and 12 months (yearly) for all the stations within ORTDM. Simple imputation method was employed to replace missing precipitation data for stations where missing data were less than 5% and observed data of neighbouring stations or reference stations were used to replace missing data where more than 5% of data were missing (Aieb et al. 2019). The yearly SPI was presented graphically to show the months which ORTDM was vulnerable to droughts throughout the referenced years of study (1998–2018). The MDM output results generated the frequency, intensity of droughts, drought durations, including minimum and maximum drought time lags. Meteorological Drought Monitor software was used to compute both the 3 months SPI moving average (3-SPI) and 6 months SPI moving average (6-SPI).

Results and findings were presented graphically using Microsoft Excel, and Microsoft Word was used to draw tables that presented the results in order to compare the outcomes across ORTDM. One-way analysis of variance (ANOVA) was used to compare SPI values and MDM output for all the local municipalities in ORTDM to uncover the ones that experienced most drought occurrences, level of severity, frequency and intensity and highlight the most vulnerable areas within ORTDM.

### Interpretation of standardised precipitation index

Negative SPI values represent rainfall deficit, whereas positive SPI values indicate rainfall surplus. The intensity of drought was classified according to the magnitude of negative SPI values such that the larger the negative SPI values were, the more serious the event was (Otkin et al. 2019). Table 2 below is an SPI interpretation and shows the level of wetness and dryness in the rainfall data of ORTDM. The findings and results of MDM output were interpreted using the SPI Table and these conformed to the SPI interpretation table.

**TABLE 2:** Standardised precipitation index interpretation table.

Interpretation	Values
Extremely wet	More than 2
Very wet	1.5–1.99
Moderately wet	To 1.49
Near normal	−0.99–0.99
Moderately dry	−1–1.49
Severely dry	−1.5–-1.99
Extremely dry	Less than -2

*Source:* Otkin, J.A., Zhong, Y., Hunt, E.D., Basara, J., Svoboda, M., Anderson, M.C. et al., 2019, ‘Assessing the evolution of soil moisture and vegetation conditions during a flash drought-flash recovery sequence over the south-central United States’, *Journal of Hydrometeorology* 20(3), 549–562. https://doi.org/10.1175/JHM-D-18-0171.1

### Ethical considerations

This article followed all ethical standards for research without direct contact with human or animal subjects.

## Results and discussions

This section presents the results of precipitation and standardised precipitation index (SPI) trends in ORTDM for the period 1998–2018. The first part of the results is the precipitation graphs for both monthly average and yearly average precipitation for the five local municipalities in the ORTDM. The second section focuses on the presentation of 3 and 6 months SPI results for ORTDM. The results are presented for 17 weather stations located across all the five local municipalities. The SPI values were further analysed to give drought severity and drought classification information for all the municipalities. Results for drought intensity include average drought intensity, maximum drought intensity, average drought duration, maximum drought duration and most intense drought duration per local municipality. The results also present areas that are vulnerable to both agricultural and hydrological droughts during the referenced period.

### Average monthly precipitation for OR Tambo district municipalities

**FIGURE 2(A – E):**
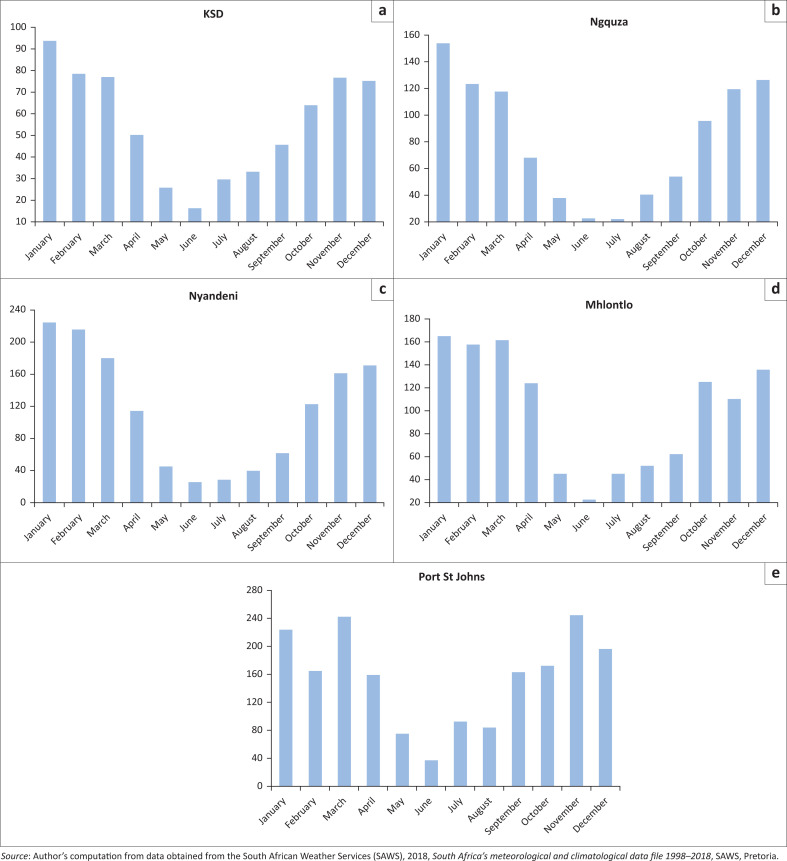
Average monthly precipitation in local municipalities of the OR Tambo District Municipality.

Figure 2A – E below depicts the average monthly precipitation for all the five local municipalities in ORTDM. The KSD municipality received its highest rainfall in summer during the months December, January and February with an average monthly precipitation of 85.6 mm. The average rainfall in summer was higher than the monthly average precipitation of 70.2 mm reported by Mditshwa and Hendrickse (2017) for the same municipality. June is reportedly the driest month in the KSD municipality and the presented results concur with the previous finding by Mahlalela et al. (2018). The KSD municipality has an annual average precipitation 68.8 mm and this is 1.1 mm less than the annual average precipitation of ORTDM.

Figure 2B depicts the average monthly precipitation patterns of Ngquza Hill for the same period (1998–2018). Ngquza Hill also receives most rainfall in December, January and February, receives the least amount of precipitation during winter (May – August) and has a monthly average precipitation of 132.7 mm and an average annual precipitation of 76.2 mm. Coastal areas along Indian Ocean are influenced by warm Mozambique current and as a result winters are wetter and warmer than the areas inland (ed. Thill 2019). Ngquza Hill is a coastal municipality and it is the second of all the local municipalities in the ORTDM that received the highest rainfall. Its average annual and average monthly precipitation was higher than the other three local municipalities in ORTDM except Port St Johns municipality. The average monthly precipitation for Port St Johns municipality is 88.0 mm and 89.9 mm is the annual average precipitation. Port St Johns municipality winters are not as dry as the rest of the other local municipalities in ORTDM and it receives sufficient rainfall and was not susceptible to hydrological drought. Nyandeni Local Municipality and Mhlontlo Local Municipality received an average monthly precipitation of 65.1 mm and 64.1 mm and average annual precipitation of 64.9 mm and 66.5 mm, respectively. They both receive extremely lower rainfall in winter. Indices used to monitor monthly trends of climate in the Eastern Cape detected that areas inland are drier and colder than coastal areas (Mahlalela et al. 2018). Mhlontlo local municipality received reduced precipitation in winter season. Mhlontlo municipality experienced its driest period in 2014 and the authorities there reported loss of livestock and reduced yields on crop production (Wambua 2019).

### Monthly standardised precipitation index and drought severity for OR Tambo District Municipality

**FIGURE 3A – E:**
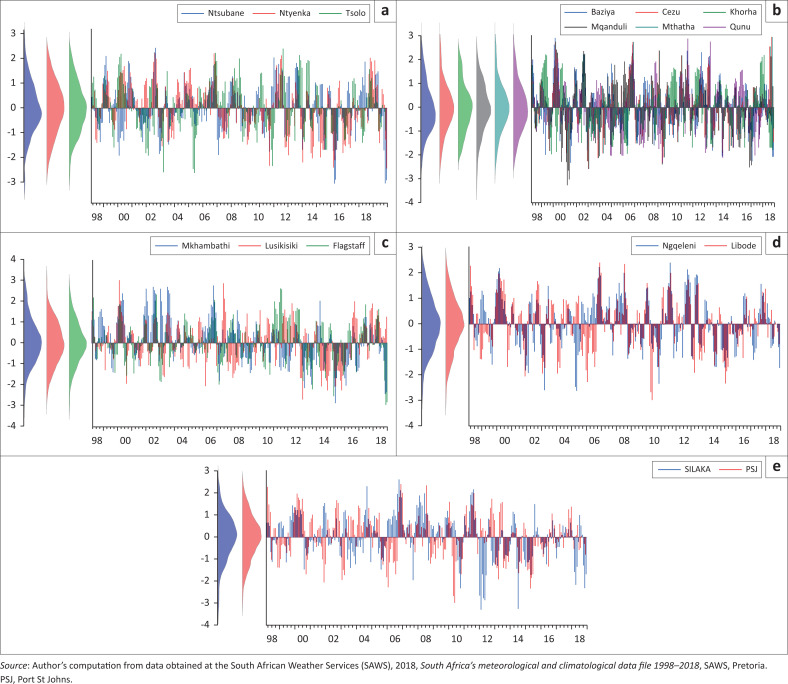
Three months standardised precipitation index and drought severity in the OR Tambo District Municipality.

Agricultural practices in ORTDM are a primary human activity; therefore, it is important to compute the 3-month SPI to reflect short- and medium-term moisture conditions that are a basis to agricultural droughts. The 3 months SPI helped to detect soil moisture, groundwater and reservoir storage (Cammalleri et al. 2019). When SPI is computed for shorter accumulation periods, in this case a period of 3 months, it is used as an indicator of reduced soil moisture and this has an important impact on agriculture and crops, especially during farming seasons. Figure 3A – E below shows the graphical representation of the 3 months SPI values distribution for all the weather stations in all the five local municipalities in the ORTDM for the period 1998–2018.

Figure 3A – E illustrates that all the five municipalities are susceptible to droughts of varying magnitude and frequency and the variation is noticeable in specific areas serviced by different weather stations. The presented graphs also show incidence of extreme to severe droughts of negative 3 months SPI values above −2.5. The incidence of meteorological, hydrological and agricultural droughts in the district municipality is not peculiar. Despite high negative drought severity figures across all the weather stations, the presented bell curves reflect higher proportion of near normal and moderately wet conditions. This is the case for all areas as per their respective weather stations. Altın, Sarış and ltın (2019) and SAWS (2018) reported similar drought patterns indicating that some parts of the country experience below-normal rainfall at varying frequencies with some areas being drier than others during the same period. These results concur with a number of previous studies in the Eastern Cape and South Africa in general (Altin et al. 2019). Mhlontlo Local Municipality is one of the driest inland municipalities in ORTDM and experienced multiple droughts negatively impacting agriculture and water sources between 1998 and 2018 (Dotse 2019). Overall, ORTDM is susceptible to droughts of varying intensity and the results of the 3 months SPI across the entire district municipality confirm this. The SPI drought categories from 1998 to 2018 of ORTDM show that some of the levels of hydrological droughts experienced have the potential to cause some devastating impact such as shortage of drinking water and reduced crop yields. Droughts negatively impact river flows, dam levels, crop yields and animal life (Jimmy et al. 2019). The presented spatial analyses of drought severity, recurrence nature of droughts and the level of variance across different areas could be used as a tool for identification of the most drought prone areas and drought periods and these could assist in resource allocation for drought preparedness.

Weather stations in the same municipalities show significant differences in SPI values. This, therefore, implies that the quality of drought data and information could be improved by increasing the density of weather stations in an area. The KSDM has the highest number of weather stations and the difference in SPI values across KSD municipality shows that the same municipality can experience varying degrees of drought in one period and this should not be generalised per municipality. A similar conclusion was made by Lucinda and Baez-Villaneuva (2019). When conducting a drought-related study, it is very important to look into the weather stations and compare the findings rather than generalising. Some critically affected areas might be overlooked because of a collective description of areas and results (Forbes & Cyr 2019).

### Months moving average standardised precipitation index for OR Tambo District Municipality (1998–2018)

The following section quantifies the amount of the time each of the five local municipalities in the ORTDM experienced drought and puts them in different categories using both 3 and 6 months SPI values. Tables 3 and 4 show 3 and 6 months SPI results for all the five local municipalities, respectively. The SPI results have been tabulated according to drought severity class of moderately dry, severely dry and extremely dry categories and computed for each municipality.

**TABLE 3:** Drought severity class table for OR Tambo District Municipality and all its local municipalities for 3 months standardised precipitation index during 1998–2018.

Drought severity class	KSD (%)	PSJ (%)	Ngquza Hill (%)	Nyandeni (%)	Mhlontlo (%)	Total % of each class in ORTDM
Moderately dry	53	21	3	21	33	32.7
Severely dry	31.6	8.7	10	8.7	14	14.6
Extremely dry	21.25	6.25	10	6.2	5.4	9.3
Total (%) of all categories drought	61.2	37	50	37	52	56.5

KSD, King Sabata Dalindyebo; PSJ, Port St Johns.

**TABLE 4:** Drought severity class table for OR Tambo District Municipality and all its local municipalities for 6 months moving standardised precipitation index during 1998–2018.

Drought severity class	KSD (%)	PSJ (%)	Ngquza Hill (%)	Nyandeni (%)	Mhlontlo (%)	Total % of each class in ORTDM
Moderately dry	53.7	14	25	20	47.1	40
Severely dry	24	7	12.5	8.3	28	22
Extremely dry	23	7	8.3	3	31	20
Total (%) of all categories drought	47.32	28.3	29.5	48	52	62

KSD, King Sabata Dalindyebo; PSJ, Port St Johns.

The summarised results in Table 3 show the summarised results per municipality. The results show that all the local municipalities in ORTDM experienced droughts of varying severity at different time proportions. The KSD Local Municipality has the highest probability of experiencing a drought with a percentage of 61.2%, followed by Mhlontlo, Ngquza Hill and lastly PSJ and Nyandeni with equal probabilities of 37%. Using the 3 months SPI values and summing up all the drought periods when the SPI is below −1, ORTDM has a 56.5% chance of experiencing drought. Agriculture is the main economic activity in ORTDM and October, November and December are the growing months. A more than 50% chance of drought in area has important implications on livelihoods and food security in areas where agriculture is the main livelihood. The higher incidence of droughts in OR Tambo was also reported by Bae et al. (2019).

In addition to results on 3 months SPI drought severity classes presented in Table 3, Table 4 presents results of the 6 months SPI for the same municipality. Unlike the 3 months SPI results which estimated a probability of 56.5% susceptibility to droughts for ORTDM, the 6 months SPI estimated a probability of 62%. Thus, the district is likely to experience more cumulative 6 months’ droughts than 3 months’ droughts. These results are in line with a study by Jimmy et al. (2019) who noted that OR Tambo DM experiences hydrological droughts that intensify into agricultural droughts in a period of 6 months. These results also confirmed reports documented by the Department of Water Affairs and Forestry (DWAF) on negative anomalies perceived in ORTDM for both on surface and subsurface water (Mditshwa & Hendrickse 2017). A report by Bae et al. (2019) highlighted only six agricultural droughts that occurred in ORTDM and these occurred in 2013, 2014, 2015, 2016 and 2017 (Bae et al. 2019). However, none of the previous studies presented drought reports according to drought severity class of moderately dry, severely dry and extremely dry categories. It is important to note that literature has vast information on droughts in the Eastern Cape and ORTDM as shown by multiple citations above but the existing information lacks some level of standardisation that can warrant comparability across both space and time. This owes to the fact that drought studies lack standardisation in terms of methodology, classification and scales used, a challenge also mentioned by Masupha and Moeletsi (2017). Widespread adoption and SPI or any methodology that promotes the calculation of the probability of droughts in any area could not be overemphasised, especially, for drought preparedness purposes.

Standardised precipitation index data and analyses characterise droughts in a way that detect both the onset and cessation of drought incidents, something that other indices are unable to do (Cook 2019). It also determines drought severity, frequency and intensity of droughts and identifies areas of high vulnerability. Information on average drought intensity and duration is crucial for decision-making purposes. Table 5 presents minimum and maximum drought intensity, average drought intensity and maximum drought duration for all the five local municipalities in ORTDM.

**TABLE 5:** Drought intensity class for OR Tambo District Municipality during 1998–2018.

Drought intensity class	KSD	PSJ	Ngquza Hill	Nyandeni	Mhlontlo
Average drought intensity	−0.77	−0.99	−0.81	−0.71	−0.62
Maximum drought intensity	−2.4 in 2014 June	−1.8 in 2014 July	−1.9 in 2015 May	−2.8 in 2014–2015 August	−3.1 in 2014 June
Average drought duration (SPI less than -1.0 for consecutive months)	1.61	1.21	1.23	2.43	2.32
Maximum drought duration	1 month, (May 2014 – June 2014)	1 month, 1 week duration (May – July 2014)	1 month, 2 weeks duration (May 2014 – July 2014)	2 months duration (May – July 2014)	2 months, 2 weeks duration (June – September 2014)
Most intense duration (DDI[M]) (SPI less than -2.0 for consecutive months)	3 weeks duration in 2014 to June 2014	5 weeks duration in 2014 (June – July 2014)	7 weeks duration (May – July 2014)	8 weeks duration (July 2014 – September 2014)	11 weeks duration (May 2014 – August 2014)

KSD, King Sabata Dalindyebo; PSJ, Port St Johns; SPI, Standardised precipitation index; DDI(M), duration of drought intensity (maximum).

Average drought intensity ranges from −0.62 to −0.99. Ngquza Hill Local Municipality is a coastal municipality. The 3 months SPI values for Ngquza Hill are in contrast to the known supposition and theory that coastal areas are wetter than inland areas (Sotsha 2013). The SPI detects the onset and end of the drought. Despite presenting a mean drought intensity that is close to normal, all the local municipalities experience long periods of drought ranging from 30 to 80 days of consecutive dry days. Mhlontlo local municipality experienced the longest drought duration of 80 days and all the municipalities experienced their longest drought duration in 2014. The results of the 2014 SPI further confirm the results by Mafongoya et al. (2019) who assert that in 2014, the whole African continent experienced its worst drought in more than 50 years with life-threatening and devastating impacts. The two coastal municipalities, PSJ and Ngquza Hill, have relatively lower average drought duration. The presented outcome confirms that Nyandeni is the driest municipality as it experiences the maximum intensity and lengthy drought duration. The results agree with the previous studies which asserted that areas inland are drier than coastal areas as noted by Mackay and Gross (2019) in drought studies conducted in Australia. In South Africa, the years 2014 and 2015 were drought years and Port St Johns Local Municipality was amongst local municipalities in ORTDM that experienced water stress and reported a reduction in water levels in its water bodies and reduced crop yields in farms (Mantsho 2018).

The SPI results could provide significant statistics that could be considered for drought monitoring, drought resources allocation and drought preparedness. When distributing drought relief aid, government departments and aid agencies should prioritise inland municipalities. In addition, these results are crucial for agricultural purpose. Accordingly, Nyandeni and Mhlontlo should be highly prioritised when it comes to agricultural drought intervention strategies such as introduction of drought tolerant plants and animals. This should be the case, especially when agriculture is a key livelihood activity. The maximum drought duration for Mhlontlo, Nyandeni and KSD in ORTDM shows that these municipalities are vulnerable and high-risk municipalities and the same was echoed by Dotse (2016). The findings of this study confirmed the hypothesis of this study that ORTDM is susceptible to hydrological droughts and furthermore, revealed the extent of drought effect, frequency, level of severity and intensity and detailed the areas of higher vulnerability.

## Conclusion

This study analyed the findings and results of drought incidents that occurred in the ORTDM during 1998–2018. The SPI output assisted in profiling and tabulating the drought incidents of ORTDM during 1998–2018, identifying the most vulnerable drought areas in ORTDM, areas of high drought intensity and most severely affected areas in the district. Average monthly precipitation for all the five local municipalities confirms that ORTDM receives more rainfall in summer than in winter and coastal areas receive high rainfall than inland municipalities. However, there are similarities in the distributions of yearly precipitation amongst all the local municipalities. All local municipalities received high precipitation during summer during the months December, January and February, and low precipitation during winter months (May, June and July). The findings of this study confirmed the hypothesis of this study that ORTDM is susceptible to hydrological droughts and furthermore, revealed the extent of drought effect, frequency, level of severity and intensity and detailed the areas of higher vulnerability. Nyandeni is the highest drought risk area in ORTDM, followed by Mhlontlo, King Sabatha Dalindyebo, Ngquza Hill and Port St Johns Local Municipality. Agricultural droughts are experienced in Nyandeni, Mhlontlo and KSDM; conversely hydrological droughts are experienced in Port St Johns Local Municipality and Ngquza Hill Local Municipality. The results could be used as a guide for drought adaptation planning and mitigation measures. The SPI could be a useful tool when forecasting and estimating the frequency, duration and intensity of droughts. However, emphasis should be placed on improving the quality of data as this is the key in improving the quality of its outcome. The generated information could add value in decision-making for the Department of Disaster Management in the ORTDM and other relevant stakeholders.
